# Research on the configurational paths for establishing high-level municipal industry-education consortiums in China: from the perspective of symbiosis theory

**DOI:** 10.1371/journal.pone.0336145

**Published:** 2026-02-27

**Authors:** Sunping Qu, Guijin Zhou, Yongmei Chen

**Affiliations:** School of Management, Wuxi University of Technology, Wuxi, Jiangsu, China; USTC: University of Science and Technology of China, CHINA

## Abstract

The municipal industry-education consortium(MIEC) is a crucial component of the development of education in China, and the provincial-level administrative regions (PARs) are committed to building high-level MIECs. However, there are significant differences in the efficiency of building MIECs in different regions across China. A province is a macro-level industry-education integration ecosystem, whereas a MIEC is a micro-level ecosystem. Symbiotic units such as industrial parks, universities, and enterprises within the provincial industry-education integration ecosystem(PIEIE) cooperate and exchange resources with each other in institutional, innovative, and digital environments to achieve the symbiotic model of industry-education integration, ultimately forming high-level MIECs. Based on the theory of symbiosis and employed the fuzzy set qualitative comparative analysis (fsQCA), this study analyzed the complex causal mechanisms through which symbiotic elements of PIEIEs influenced the construction of high-level MIECs, using data from the 31 PARs in China, excluding Hong Kong, Macao and Taiwan. This paper found that the development of high-level MIECs was not determined by any single symbiotic element; instead, it resulted from the coordinated development and combined effects of three key symbiotic factors: symbiotic units, symbiotic environment, and symbiotic models. There were six configurational pathways to building high-level MIECs, grouped into three types: the “Economy-Driven” model, the “Digital-Enabled Industry-Education Integration” model, and the “Assistance-Driven” model. The findings provide a theoretical foundation and practical guidance for PARs in developing high-level MIECs.

## 1. Introduction

Industry-education integration is an important step in the reform of vocational education. On the one hand, it provides vocational colleges and universities with teaching, learning, and research resources, thereby enhances the capacity of vocational education [1]; on the other hand, it supplies industries with technological innovation and human resources, fostering socio-economic development [2]. The municipal industry-education consortium (MIEC) is an important initiative of industry-education integration, which refers to an entity that is based on industrial parks, integrates educational and industrial resources, and aims to promote the high-quality development of education and industry. There are four stages to promote MIECs in China. The first stage is the nascent phase. In 2022, the Central Committee of the Communist Party of China and the State Council issued *The Opinions on Deepening the Reform of the Modern Vocational Education System and Structure*, proposing to take industrial parks as the foundation and build MIECs that integrate talent cultivation, innovation and entrepreneurship, and the promotion of high-quality industrial economic development. The second stage is the initial implementation phase. In 2023, the Ministry of Education issued *The Notice on Carrying out the Construction of MIECs*, clarifying the construction goals of MIECs from 2023 to 2025, and released *The Indicators for the Construction of MIECs*. In September 2023, the Ministry of Education announced the first batch of 28 national MIECs. The third stage is the deepening stage. In 2024, the Ministry of Education issued *The Notice on Strengthening the Construction of MIECs*, further clarifying the construction content of MIECs, and announcing the list of the second batch of 6 national MIECs. In addition, *The Construction Standards for MIECs* was also promulgated as a supporting measure. The fourth stage is the upgrading phase. In 2025, the Communist Party of China Central Committee and the State Council jointly issued *The 2024–2035 master plan on building China into a leading country in education*, which stated that China should strengthen MIECs, and optimize the layout of vocational education that is coordinated with regional development and compatible with industry. So far, the importance of MIECs has elevated from a component of vocational education reform to a key part of the strategy for building a modern education nation.

In China, all provincial-level administrative regions (PARs) place great importance on MIECs and have issued a series of institutional incentives and guidelines to guide the construction of MIECs. However, there are significant differences in the development of MIECs across different regions. Taking national MIECs as an example, the Ministry of Education had announced the list of two batches of national MIECs, totaling 34, by December 2025. In terms of geographical distribution, these consortia show significant imbalances among the 31 PARs in China, excluding Hong Kong, Macao and Taiwan. Jiangsu Province, which has four national MIECs has become the province with the most national MIEC, followed by Zhejiang Province and Sichuan Province, which have three national MIECs. In contrast, nine provincial-administrative regions have no national MIEC. Why do the numbers of national MIECs vary so significantly across regions? There is no difference in policy support at the national level, so the gap must exist in the 31 PARs. The 31 PARs have different degrees of educational and industrial resources, uneven levels of integration of industry and education, which have resulted in the imbalance among regions in the construction of high-quality MIECs.

From the perspective of ecosystems, PARs are regarded as regional industry-education integration ecosystems (RIEIEs), in which MIECs grow and develop. Ecosystems originated in the field of biology and were later applied to the economic and social fields [3].The symbiosis theory of the ecosystem reflects the interrelationships between different symbiotic units in the ecosystem, and between symbiotic units and symbiotic environment, and is an essential theoretical basis for the construction of high-level MIECs, which is suitable for analyzing the complex mechanisms of the operation and construction of MIECs [4]. Previous studies have used symbiosis theory to analyze the construction mode and paths of MIECs, as well as the challenges encountered by MIECs and the countermeasures to address the challenges [4–6], and analyzed the role of digital technologies in the construction and governance of MIECs [7]. However, existing research has not adequately explained why there is such a large gap in the number of national MIECs across various PARs.

Existing studies are not good enough to explain the differences in the construction of MIECs across regions. First, Most existing literature has rarely examined the construction of MIECs from a provincial perspective. Second, existing research methods were dominated by qualitative descriptions, with little data-based quantitative validation, making it difficult to accurately identify the key combinations of conditions that drive the formation of high-level MIECs. Third, existing research focuses on the independent influence of single elements (e.g., institutional environment, enterprise participation), but neglects the complex mechanism of the interaction of the symbiotic elements of “symbiotic unit-symbiotic mode-symbiotic environment”.

In view of this, this study takes the symbiosis theory as the core analytical framework and adopts the fuzzy set qualitative comparative analysis (fsQCA) method to analyze the empirical data from the 31 PARs in China. Specifically, this study aims to address three key research questions:(1) What is the current status of the construction of high-level MIECs across the 31 PARs in China? The Ministry of Education has published the official list of national MIECs, enabling researchers to identify how many high-level MIECs are located in each of the 31 PARs. However, the 31 PARs differ substantially in scale—such as population size—so a simple count of high-level MIECs is insufficient to capture the true extent or intensity of MIECs development in each region. To account for demographic differences, this study compares MIECs development across provinces on a per capita basis, using population as a key normalization factor. (2) What factors in PARs influence the development of MIECs in PARs, and through what mechanisms do these factors operate? Although existing literature has examined the operational status of MIECs, the challenges they commonly face, and corresponding policy responses, few studies have systematically investigated— from a provincial perspective—the key symbiotic elements that shape the formation of high-level MIECs and the pathways through which they exert their effects. (3) What strategies can be adopted to facilitate the construction of high-level MIECs in PARs? As MIECs are policy-driven institutional outcomes shaped by multi-level policy frameworks, analyzing the development of high-level MIECs from a provincial-level perspective is highly feasible and policy-relevant.

## 2. Literature review and theoretical framework

### 2.1. Literature review

Industry-education integration is a symbiotic development of the industrial system and the education system based on shared values, with the collaboration of stakeholders such as the government, enterprises and universities through in-depth integration [8–10]. The MIEC is a vital policy top-level design of the Chinese government to promote the integration of industry and education. Han et al. (2023) pointed out that a MIEC refers to a new form of industry-education integration organization that is led and promoted by the government within the jurisdiction of a prefecture-level city or a prefectural-level administrative region with legislative power. It is based on a particular industrial park as a platform, integrating various cooperative entities such as universities, industry enterprises, research institutions, and social organizations (including financial institutions and service institutions), and forming a new type of industry-education integration organization through close contractual relationships [11]. Xu et al. (2025) argue that a MIEC is an entity organization based on an industrial park that possesses leading industries such as advanced manufacturing, modern service industry, and modern agriculture. It is jointly constructed by multiple entities including the government, industry leaders, vocational colleges, and research institutions [12]. It integrates various resources such as funds, technology, talents, and policies, and has dual functions of talent cultivation and industrial service [13]. In essence, a MIEC is a kind of regional industry-education integration platform with multi-party cooperation and resource sharing, which has the characteristics of regionality, openness, plurality, ecology and complexity [11,12].

MIECs have three characteristics. First, regional coordination and government-led: MIECs are usually led by prefecture-level cities, which offer support policies such as land, finance, taxation, and talent. They integrate resources from industrial parks, universities, and enterprises within the city, conduct overall planning, and promote the development of MIECs in a coordinated manner, breaking through the limitations of previous point-to-point school-enterprise cooperation and project-based cooperation. Second, focusing on the needs of municipal industrial development, with mutual benefit between industry and education: MIECs are closely aligned with the leading industries, strategic emerging industries, and future industries. They develop technologies and cultivate talent, realizing mutual benefit between universities and enterprises and promoting the high-quality development of local industry-education integration. Third, operationalization: MIECs ensure their effective operation by establishing a three-tier structure consisting of a Council, a Secretariat, and member liaison offices [4,14,15].

Symbiosis theory is an essential foundational theory for the regional industry-education integration ecosystem (RIEIE). The theory of symbiosis was proposed by the German botanist Heinrich Anton De Bary in 1879. It was initially applied in the biological field for studying the coexistence of different species [16], and later was employed in the social sciences to study cross-border cooperation and coordinated development among different organizations [17]. Wu et al. (2023) argued that using symbiosis theory to analyze MIECs is highly applicable and operable [4]. Some scholars have studied the operational mechanism of the MIECs from the perspective of symbiosis theory, including policy guidance mechanism, industry-education integration mechanism, benefit sharing mechanism, performance evaluation mechanism and comprehensive governance mechanism [12]. They believed that MIECs encountered several challenges during their development, such as an incomplete symbiotic unit integration mechanism, poor collaboration; slow evolution of the mutually beneficial symbiotic model, and inadequate information linkage, weak ecological environment construction, and a lack of adequate policy support that can be implemented [5,13]. Scholars have made recommendations, such as improving the collaborative governance mechanism, establishing an integrated symbiotic model and a symmetrical mutual benefit symbiotic model, optimizing the symbiotic environment, and enhancing the effectiveness of cooperation [4].

In summary, existing literature mainly studies what MIECs are from a micro perspective, how participants within MIECs cooperate with each other from a symbiotic perspective, the operational problems of MIECs, and the corresponding solutions.

### 2.2. Theoretical framework

Ecosystem theory provides an integrated and dynamic framework for research, emphasizing the interdependence among constituent actors and their interactions with the broader environment. Studying MIECs through an ecosystem lens offers two key advantages. First, it overcomes a major limitation of traditional competition-based theories, which focus narrowly on the survival and growth of individual organizations while neglecting the roles of other stakeholders. Second, it avoids overly simplistic prescriptive approaches that propose idealized models of how MIECs “should be” without accounting for the behavioral mechanisms of actors, their mutual interactions, or the influence of environmental contexts. As a result, the ecosystem perspective enables a more scientific, holistic, and actionable understanding of MIECs development, thereby enhancing both its theoretical depth and practical relevance for promoting sustainable collaboration.

MIECs grow and develop in PIEIEs. The three symbiotic elements of PIEIEs - symbiotic unit, symbiotic environment, and symbiotic mode – play a critical role in shaping MIECs.

#### 2.2.1. The impact of symbiotic units on high-level MIECs.

Like natural ecosystems, the symbiotic units of PIEIE have diversity, population competition, community order and system adaptability. Information exchange, energy flow and material circulation between symbiotic elements form an ecosystem of symbiotic competition and dynamic evolution. There are three core members in PIEIE ——industrial parks, enterprises and universities. Industrial parks include new districts, economic development zones, high-tech industrial parks, and airside economic zones. Enterprises are mainly those in the industrial parks. Universities include higher education institutions such as vocational colleges and universities.

**2.2.1.1 Industrial park:** As a coordinating body, industrial parks are the foundation for the establishment of the MIEC, and the management committee of the industrial park, which is a dispatching or special administrative organ of the local government or a higher-level government, need to make a good top-level design for the formation and development of the MIECs according to the regional development strategy and the foundation of the industrial park. The role of the industrial park is mainly in the following three aspects: Firstly, the industrial park determines the development direction of the MIECs according to the leading industries and the strategic emerging industries in the industrial park. Secondly, the industrial park takes the lead in setting up the board of directors of the municipal industry-education consortium, which is composed of the local government where the industrial park is located, enterprises, universities and other units, so as to improve the cooperation mechanism of the MIEC. Thirdly, the industrial park introduces detailed measures to clarify financial, fiscal, tax, land, credit, employment and other incentives, so as to provide policy support for the MIECs.

**2.2.1.2 University:** As the main body of talent training and technology supply, universities should not only be rooted in the region, integrated into the industry, and implement the fundamental task of vocational education to cultivate morality and nurture people, but also make use of their scientific research capabilities and human resources to provide technological innovation and consulting services for enterprises. The first is to provide technical and skilled talents for enterprises and strengthen their human resources. The second is to provide job training for enterprise employees, providing pre-employment training, job training and continuing education. The third is to provide technical services for enterprises and help them transform and upgrade.

**2.2.1.3 Enterprise:** As the main driving force, enterprises upstream and downstream of the regional industrial chain play the role of enterprises participating in education, and cooperate in the construction of curricula, teaching materials and teachers, etc., so as to promote the integration of industry and education. Firstly, they are deeply engaged in cooperation in running universities, building industrial colleges in cooperation with universities, and building practical training bases for the integration of industry and education. Secondly, they are deeply engaged in cooperation in education, providing resources and an environment for talent cultivation. Thirdly, they are deeply engaged in cooperative development, relying on the consortium to build a regional technology innovation centre and transformation centre, realizing the transformation and upgrading of enterprises, and providing impetus for the high-quality development of the regional economy.

#### 2.2.2. The impact of symbiotic environment on high-level MIECs.

The stable operation of MIECs is premised on a perfect ecological environment. Chen et al. (2024) argue that the symbiotic environment of MIECs mainly includes the innovation environment and the institutional environment [18]. As the digitisation of society and economy continues, the application of digital technology in education and industry is becoming more and more widespread, facilitating the integration of industry and education [19]. Therefore, this paper analyzes the symbiotic environment of MIECs from three aspects: innovation environment, institutional environment and digital environment.

**2.2.2.1 Innovation environment:** The innovation environment usually refers to the overall conditions and factors that support and promote innovation activities. It is not only a key component in the external environment of the MIECs, but also one of the core driving forces for its sustainable development. MIECs have the functions of talent cultivation, innovation and entrepreneurship, and promotion of high-quality development of industrial economy, and they promote technological innovation, process improvement, and product upgrading through the construction of common technical service platforms, and provide technical consulting and services for enterprises in the industrial park. An excellent innovation environment can enhance the willingness of the participating units of MIECs to carry out industry-education integration, expand the investment in industry-education integration, and then enhance the construction of MIECs.

**2.2.2.2 Institutional environment:** The institutional environment refers to the external institutional factors that shape an organization and its behavioral patterns. According to Scott’s three-dimensional model of institutional environment, the institutional environment contains three dimensions: mandatory factors, normative factors, and cognitive factors [20]. Mandatory factors include national mandatory laws and regulations, government administrative orders, systems, policies, etc., which play a guiding role in the direction, scope, and implementation mode of industry-education integration. The government has introduced “policy packages” to support the sustainable development of MIECs, such as tax incentives, financial subsidies and other policies to encourage enterprises to participate in talent cultivation, and the introduction of relevant laws and regulations to protect the rights and safety of students during their internships. All these are conducive to the long-term development of industry-education integration and provide a policy guarantee for industry-education integration. Normative factors include social default codes of conduct, norms, standards, etc., which can guide universities to meet market demand, and also help enterprises to perceive their social responsibility to participate in education. When universities and enterprises generally accept university-enterprise cooperation, it will be conducive to the development of industry-education integration. Cognitive factors include taken-for-granted cultural identity, values, etc., which can help enterprise managers realise that university-enterprise cooperation can enhance the skill level of their employees and strengthen the innovation ability of their enterprises, and also university teachers and students can realise the importance of enterprises’ participation in education. Such a consensus reached by all parties is conducive to overcoming obstacles in cooperation and promoting the implementation of the results of the industry-education integration project. Therefore, a good institutional environment is conducive to the creation and development of MIECs.

**2.2.2.3 Digital environment:** In the digital era, leveraging digital technologies to build a collaborative platform enables MIECs participants to exchange information efficiently and at low cost, and to share information and resources—thereby facilitating the development of high-level MIECs [21]. First, focusing on technology empowerment, building a community platform for industry-education integration with shared resources. The second is to improve the operation of the entity, and promote the same direction of the participating units. The third is to explore the construction of long-term mechanism to create an ecological environment of mutual integration and coexistence. For example, in order to improve the function of the MIECs in bringing together industry-education resources, the national MIEC, Wuxi Integrated Circuit Industry-Education Consortium, has procured the software of the entity-based platform of the industry-education consortium and the platform operation and data service, of which the software part includes the membership of the MIEC and the management of the project, the collection of data of the MIEC, the release of information on the integration of industry and education, the docking of supply and demand between the industry and education, analysis of big data of industry and education and the management of the development of personnel training and other six modules, so as to achieve a high degree of matching between industry and education and efficient services

#### 2.2.3. The impact of the symbiosis model on high-level MIECs.

The symbiotic model reflects the symbiotic state of multiple agents in an ecosystem [22]. The higher the level of the symbiosis model, the higher the possibility of forming a high-level MIEC. In a MIEC, enterprises and universities have a strong complementarity in terms of resources, capabilities and demands. On the one hand, the symbiotic units in the MIECs can enhance communication and reach consensus through mutual collaboration, thus reducing the risks in university-enterprise cooperation. On the other hand, the symbiotic units in the MIECs can facilitate the provision of complementary resources and capabilities between them, compensating for the lack of resources and capabilities of the symbiotic units [23].

#### 2.2.4. The impact of symbiotic elements on high-level MIECs under the configurational perspective.

The evaluation of high-level MIECs is implemented by the Ministry of Education. The construction requirements for high-level MIECs are comprehensive and quantitative, imposing requirements on symbiotic units, symbiotic environments, and symbiotic cooperation models. The Ministry of Education issued the Construction Indicators for MIECs in 2023 and the Interim Construction Standards for MIECs in 2024 respectively. The latter released in 2024 is more detailed. According to the perspective of construction criteria, the evaluation of national-level MIECs constitutes a comprehensive evaluation. The evaluation indicators are divided into three types: fundamental indicators, substantive indicators, and negative indicators. Fundamental indicators set requirements for the foundations of provincial governments, municipal governments, industrial parks, universities, and enterprises. For instance, they assess whether provincial and municipal governments have issued policy measures to support the development of MIECs or convened dedicated meetings to advance MIECs construction; whether the industrial park is designated as a national-level new area or economic development zone; whether the lead enterprise is an industry leader or a “specialized, sophisticated, and innovative” (“Little Giant”) enterprise; and whether the lead university is a nationally or provincially recognized high-performing vocational college or part of a high-level vocational program cluster. Substantive indicators specify required actions and expected outcomes across four dimensions: collaborative education delivery, joint talent cultivation, cooperative employment support, and co-development initiatives. For example, they assess whether a governing council has been established; whether financial, fiscal, tax, land-use, and credit-based incentive policies have been provided; how many national-level courses, national-level planned textbooks, additional training bases, and digital teaching resources have been co-developed; what the employment rate, employment alignment rate, and local employment rate of students are; how many patents have been provided to enterprises, how many employees have been trained, and the total value of technical service contracts signed. Negative indicators specify the fundamental errors that cannot be committed in MIECs, such as major illegal or disciplinary incidents occurring in enrollment, employment, safety, and other areas; the failure to disburse planned investment funds; and major fraud in the application and construction process.

The construction of high-level MIECs is often not the result of the separate influence of a single symbiotic element, but the result of the joint action of multiple symbiotic elements [24]. Firstly, the establishment of high-level MIECs imposes requirements on symbiotic units and requires the concerted efforts of these units. Secondly, the completion of the construction tasks of high-level MIECs imposes requirements on the symbiotic environment. Participating parties (PARs) need an institutional environment with sound incentives such as financial, fiscal, tax, land, and credit policies, an innovative environment that encourages cooperation and innovation among universities, enterprises, and research institutions, and a digital environment that facilitates school-enterprise cooperation. Finally, the completion of the construction tasks of high-level MIECs also imposes requirements on the symbiotic model. Industrial parks, universities, and enterprises need to cooperate in education, talent development, employment, and industrial development. Therefore, it is necessary to analyze the influence of symbiotic elements on the creation of high-level MIECs from a configurational perspective.

**Fig 1:**
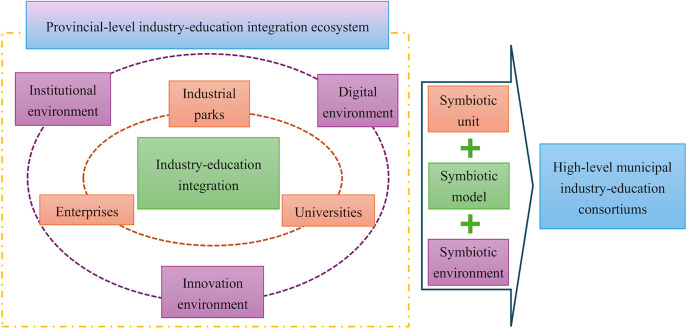
Creation of high-level MIECs from configurational perspective.

In summary, as shown in Fig 1, drawing on symbiosis theory and adopting a configurational perspective, this study explores the configurational pathways through which the symbiotic elements of PIEIEs promote the high-quality development of MIECs. It aims to address the central research question: “How can the configuration of symbiotic elements within the PIEIEs be optimized to achieve the high-quality development of MIECs?”

## 3. Research methodology and data construction

### 3.1. Research methodology

The research method used in this paper is fsQCA [24]. fsQCA combines the advantages of qualitative and quantitative analyzes by treating the result variable as a result of the grouping of antecedent variables, and is commonly used to analyze causal complexity problems such as multi-causal concurrency, causal asymmetry and equifinality [25]. The use of fsQCA to explore the complex causal mechanisms of high-level MIECs is based on the following three considerations. First, the creation of high-level MIECs is often the result of multiple factors within the PIEIEs, which is neither determined by a single condition nor by multiple conditions separately. Second, the fsQCA method can effectively analyze the interaction of three or more variables, so the process can well analyze the creation of high-level MIECs. Third, the fsQCA method suggests a sample size between 30 and 1000, which is suitable for small samples. The sample size in this paper is 31, which is suitable for fsQCA [26].

### 3.2. Variable measurement and data sources

#### 3.2.1. Result variable.

An important representative of high-level MIECs is the national MIECs. In this paper, the number of national MIECs in the 31 PARs is chosen to measure the creation of high-level MIECs. The Ministry of Education announced two batches of 34 national MIECs in 2023 and 2024. Considering the differences in the scale of the 31 PARs, this study normalizes the count of national MIECs in each province by dividing it by the province’s population(in hundreds of millions), thereby removing the impact of provincial scale disparities.

#### 3.2.2. Conditional variables.

According to the symbiosis theory, the symbiosis three elements of the RIEIE mainly include the symbiosis unit, the symbiosis mode and the symbiosis environment. Symbiotic units include universities, enterprises and industrial parks. The symbiosis mode is measured by industry-education synergy. The symbiotic environment includes innovation environment, institutional environment and digital environment.

Industrial park: National High-Tech Industrial Development Zones (HTZs) and Economic and Technological Development Zones (ETDZs) serve as a key indicator of industrial park development in the region. In this paper, we use the count of national HTZs and ETDZs within each PAR to gauge the development level of major industrial parks in the region, with data sourced from two official lists: *the List of National ETDZs* published by the Ministry of Commerce of the People’s Republic of China, and *the List of national HTZs* released by the Ministry of Science and Technology of the People’s Republic of China. To eliminate the confounding impact of scale differences across China’s 31 PARs—consistent with the data normalization approach applied to other indicators in this research—this study divides the total number of HTZs and ETDZs in each region by its permanent population size.

**3.2.2.1 University:** A key indicator of advanced educational development lies in the number of universities situated within a region. In this study, the number of general higher education institutions in each PAR is employed to gauge the regional university development level, with data sourced from *the List of National Higher Education Institutions* (as of 15 June 2023) released by the Ministry of Education in China. Given the substantial variations in the scale of China’s 31 PARs, this study normalizes the count of higher education institutions in each province by dividing it by the province’s population, thereby eliminating the confounding impact of regional scale disparities on the measurement results.

**3.2.2.2 Enterprise:** The number of enterprises serves as a critical indicator of the development level of the industry-education integration ecosystem. In this study, the count of enterprises across China’s 31 PARs in 2023 is used to measure the development level of enterprise entities, drawing on official statistics from the *Communiqué on the Fifth National Economic Census of the People’s Republic of China*. For the purpose of removing the confounding effect of provincial scale disparities, this study normalizes the count of legal entities in each PAR by dividing it by the region’s population.

**3.2.2.3 Innovation environment:** The regional innovation environment score is used to measure the innovation environment, and the data source is *China Regional Science and Technology Innovation Capacity Evaluation Index Report (2023)*.

**3.2.2.4 Institutional environment:** The regional marketability index is used to measure the institutional environment, and the data source is *the China Sub-Provincial Business Environment Index 2023 report* published by the China Marketization Index database.

**3.2.2.5 Digital environment:** The digital economy index is a key indicator of the digital environment. In this study, we adopt *the China Digital Economy Index (2024)* released by the Beijing Institute of Mathematical Sciences and Applications (https://bimsa.net/doc/publication/15683.pdf), which provides provincial-level digital economy index values for 2023. However, the digital environment is influenced by both the innovation environment and institutional environment, resulting in a high correlation between the digital economy index, market index, and innovation index—and thus giving rise to multicollinearity among these indicators. To address this issue, this study normalizes the provincial digital economy index by dividing it by the regional population size, thereby eliminating the scale effect and reducing the degree of correlation among the indicators.

**3.2.2.6 Industry-education integration:** Regional industry-education integration reflects the degree of the industry-education integration symbiosis model. In this study, we adopt the findings of Zong et al. (2024) to measure the symbiosis model [27], who calculated provincial scores for the quality of industry-education integration based on *the 2023 China Vocational Education Quality Annual Report*.

## 4. Results

This study employed fsQCA 3.1 to analyze the configurational paths for establishing high-level MIECs. fsQCA analysis includes four steps: data calibration, Necessity analysis of individual condition, Configuration analysis and Robustness check.

### 4.1. Data calibration

Data calibration assign a specific degree of affiliation to each case. Referring to the study of Sun Yanfang et al. (2025) and combining the characteristics of the cases in this paper, three values of 85% quartile (full membership), 50% quartile (crossover point) and15% quartile (full non-membership) are set as the calibration points. When the fuzzy affiliation score of the calibrated case is exactly 0.5, it can lead to sample loss. To avoid this, the value of 0.5 is corrected to 0.499 in this paper. The fundamental situation of the variables is shown in Table 1.

**Table 1:** Descriptive statistics of variables and data calibration.

Variables			Mean	S.D	Min	Max	Fuzzy set calibration		
							Full non-membership	Crossover	Full membership
Result variable	MIEC		2.519	2.791	0.000	14.660	0.000	2.391	4.326
Conditional variables	Symbiotic unit	Industrial park (P)	31.579	13.448	9.150	54.790	17.095	30.364	50.132
		University (U)	225.081	64.049	127.500	420.860	169.829	210.930	275.172
		Enterprise (E)	223.108	90.973	120.510	539.340	146.286	202.218	317.458
	Symbiotic environment	Innovation environment (IE)	68.309	8.147	56.520	95.230	60.414	66.860	76.464
		Institutional environment (InstE)	7.625	2.043	3.330	10.720	5.312	7.940	9.806
		Digital environment (DE)	90.090	72.280	27.150	292.050	41.541	59.818	194.844
	Symbiotic model	Industry-education integration (IEI)	44.667	8.245	19.290	66.350	38.460	43.370	52.562

### 4.2. Necessity analysis of individual condition

Before configuration analysis, we should analyze whether an individual variable serves as a necessary condition for establishing high-level or non- high-level MIECs. The results of the necessity analysis are shown in Table 2. If the consistency value of a condition is greater than 0.9, the condition is a necessary condition for the outcome. In Table 2, the consistency of all conditions that led to high-level and non-high-level MIECs are below 0.9, so there is no necessary condition which causes either high-level or non-high-level MIECs.

**Table 2:** Necessity test for single condition.

Conditional Variables	Result Variables			
	high-level MIECs		non-high-level MIECs	
	Consistency	Coverage	Consistency	Coverage
P	0.672	0.682	0.516	0.540
~P	0.547	0.522	0.696	0.687
U	0.662	0.651	0.543	0.552
~U	0.544	0.536	0.656	0.667
E	0.634	0.675	0.480	0.528
~E	0.556	0.509	0.704	0.665
IE	0.691	0.690	0.521	0.537
~IE	0.536	0.520	0.699	0.700
InstE	0.692	0.677	0.503	0.508
~InstE	0.497	0.492	0.680	0.695
DE	0.575	0.638	0.559	0.640
~DE	0.675	0.597	0.684	0.624
IEI	0.675	0.663	0.530	0.537
~IEI	0.528	0.521	0.667	0.680

### 4.3. Configuration analysis

After conducting a necessity analysis of individual conditions, this paper employed fsQCA 3.1 to perform configurational analysis. Because the total sample size was only 31, we set the case frequency threshold at 1, meaning that each configuration had to be supported by at least one observed case [28]. Based on the actual distribution of raw consistency values, a precise breakpoint was observed at 0.79. Therefore, we set the raw consistency threshold at 0.79. Following conventional recommendations, we set the PRI consistency threshold at 0.7 [28]. fsQCA provides three types of solutions——complex solution, intermediate solution and parsimonious solution. We integrated the results of the intermediate and parsimonious solutions, treating conditions in the parsimonious solution as core conditions and those in the intermediate solution as edge conditions. The configurations consist of both core and edge conditions: core conditions are the essential drivers that are necessary for achieving the expected outcome, while edge conditions play a supplementary or supportive role. The configurations leading to high-level MIECs are presented in Table 3.

**Table 3:** Configurations for high-level MIECs.

Variables		H1a	H1b	H2a	H2b	H2c	H3
Symbiotic unit	P	●	●	•	•	ⓧ	●
	U	ⓧ	ⓧ	•		•	•
	E	•	•	•	•	•	ⓧ
Symbiotic environment	IE	●	●		•	•	ⓧ
	InstE	●	●	•	•		ⓧ
	DE	ⓧ		●	●	●	ⓧ
Symbiotic model	IEI		•	●	●	●	ⓧ
Consistency		0.981	0.980	0.986	0.987	0.954	0.908
Raw Coverage		0.273	0.290	0.283	0.300	0.245	0.214
Unique Coverage		0.017	0.028	0.022	0.000	0.083	0.100
Solution Consistency		0.948					
Solution Coverage		0.633					

Note: ● indicates that the core condition exists, • indicates that the edge condition exists, ⓧ indicates that the core condition is missing, ৹ indicates that the edge condition is missing. The same notation applies below.

As shown in Table 3, there are six configurations leading to high-level MIECs. Following the principle of grouping configurations that share the same core conditions, six configurations can be classified into three types. In configuration H1, P, IE, and InstE are present as core conditions, whereas U is absent as a core condition. In configuration H2, DE and IEI are present as core conditions. In configuration H3, P is present as a core condition, whereas IE, InstE, DE, and IEI are absent as core conditions.

In order to test causal asymmetry, this study analyzed the configurations of symbiotic systems that produce non-high-level MIECs. As shown in Table 4, there are four configurations leading to non-high-level MIECs, which can be grouped into three types. In configurations L1, U and InstE are absent as core conditions, while DE is present as a core condition. In configurations L2, P and IEI are absent as core conditions, while U is present as a core condition. In configurations L3, P and IEI are absent as core conditions, while IE is present as a core condition. The consistency of all configurations is greater than 0.8, meeting the consistency threshold. The overall consistency is 0.948, ensuring the reliability of the results [28].

**Table 4:** Configurations for non-high-level MIECs.

Variables		L1a	L1b	L2	L3
symbiotic unit	P	•	৹	ⓧ	ⓧ
	U	ⓧ	ⓧ	●	৹
	E		৹	৹	•
symbiotic environment	IE	৹		৹	●
	InstE	ⓧ	ⓧ	৹	•
	DE	●	●		৹
symbiotic model	IEI	৹	৹	ⓧ	ⓧ
Consistency		0.961	0.974	0.916	0.940
Raw Coverage		0.203	0.215	0.230	0.149
Unique Coverage		0.091	0.047	0.069	0.051
Solution Consistency		0.907			
Solution Coverage		0.434			

### 4.4. Robustness check

Results are considered robust if slight changes in the operational parameters do not substantially alter the outcomes. In order to verify whether the configurations for high-level MIECs are robust, this paper adjusted the parameters in three different ways. First, keeping all other parameters unchanged, we increased the raw consistency threshold from 0.79 to 0.85 ro 0.90 and the results remained completely unchanged, because the consistency cutoff is 0.908. Second, with all other parameters held constant, we raised the PRI threshold from 0.7 to 0.8. The results are presented in Table 5. Because the configuration involving Xinjiang case has a PRI of 0.792, it no longer meets the new threshold and is thus excluded from the pathways. However, the other two types of configurations did not undergo any substantial changes. Third, again keeping all other parameters fixed, we adjusted the full membership threshold from 0.85 to 0.8 and the full non-membership threshold from 0.15 to 0.20. The results are also presented in Table 5. None of the three pathway types exhibited any substantial changes.

**Table 5:** Results of Robustness check.

Robustness check	Variables		T1	T2	T3	T4	T5
Change PRI threshold from 0.7 to 0.8	Symbiotic unit	P	●	●		•	•
		U	ⓧ	ⓧ	•	•	
		E	•	•	•	•	•
	Symbiotic environment	IE	●	●	•		•
		InstE	●	●	●	●	●
		DE	৹		●	●	●
	Symbiotic model	IEI		•	•	•	•
	Consistency		0.981	0.980	0.986	0.986	0.987
	Raw Coverage		0.273	0.290	0.331	0.283	0.298
	Unique Coverage		0.019	0.028	0.076	0.027	0.000
	Solution Consistency		0.980				
	Solution Coverage		0.516				
Change threshold for member from 0.85 to 0.8, and threshold for non-member from 0.15 to 0.2.	Symbiotic unit	P	●	•		●	
		U	ⓧ	•	•	•	
		E	•	•	•	৹	
	Symbiotic environment	IE	●		•	ⓧ	
		InstE	●	●	●	ⓧ	
		DE		●	●	ⓧ	
	Symbiotic model	IEI	•	•	•	•	
	Consistency		0.991	0.988	0.990	0.914	
	Raw Coverage		0.278	0.265	0.326	0.218	
	Unique Coverage		0.150	0.015	0.091	0.129	
	Solution Consistency		0.960				
	Solution Coverage		0.636				

## 5. Discussion and implications

### 5.1. Discussion of the key findings

#### 5.1.1. Distribution of high-level MIEC.

**Fig 2:**
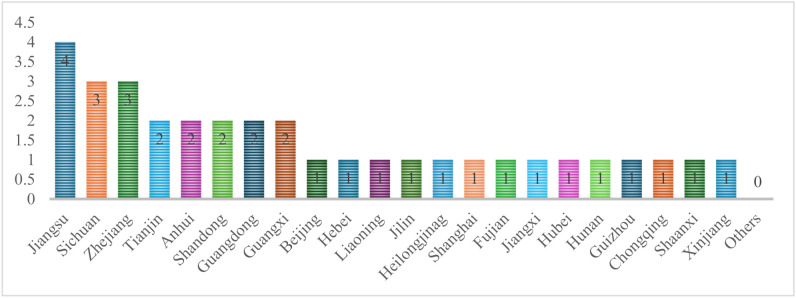
Number of national MIECs in each region.

As shown in Fig 2, the number of national MIECs owned by the 31 PARs falls into five tiers: Jiangsu leads with four; Sichuan and Zhejiang follow with three each; Tianjin, Anhui, Shandong, Guangdong, and Guangxi, which own two; then Beijing, Hebei, Liaoning, Jilin, Heilongjiang, Shanghai, Fujian, Jiangxi, Hubei, Hunan, Guizhou, Chongqing, Shaanxi, and Xinjiang, with one each; and finally Henan, Yunnan, Gansu, Qinghai, Ningxia, Inner Mongolia, Tibet, Hainan, and Shanxi, which are the nine regions without any national MIEC.

In terms of geographical distribution, the PARs with more national MIECs are mainly located in the southeast coastal region in China, and most of these PARs have more than two national MIEC; followed by PARs with one national MIEC, which are located in the northeast and the south-central region; and lastly PARs without national MIEC, which are distributed in the northwestern region. It can be seen that the distribution of high-level MIECs is closely related to the level of economic development of the region, and economically developed PARs generally have more national MIECs.

**Fig 3:**
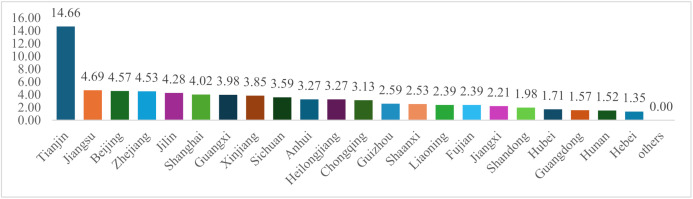
Number of national MIECs adjusted for regional population in each region.

Fig 3 illustrates the development status of national MIECs, adjusted for regional population. The vertical axis represents the number of national MIECs in each region divided by the region’s population (in hundreds of millions). As shown in Fig 3, Tianjin exhibits an exceptionally high value, primarily because it hosts two national MIECs despite its relatively small population. It is followed by Jiangsu, Beijing, Zhejiang, and other regions. Although Guangdong and Shandong each have two national MIECs, their large populations—ranking first and second among the 31 PARs—result in lower rankings when adjusted for population.

#### 5.1.2. Mechanism analysis of high-level MIECs.

Three types of configurations lead to high-level MIECs. Configuration H1, which we name the “Economy-Driven” model, is primarily represented by provinces such as Jiangsu, Zhejiang, and Anhui. These provinces feature favorable institutional and innovation environments, strong economic development, and well-established industrial parks and enterprises. Their DE ranks at a moderate level (Zhejiang: 12/31, Jiangsu: 18/31, Anhui: 19/31). Although their per capita higher education resources are relatively low (Anhui: 21/31, Jiangsu: 22/31, Zhejiang: 28/31), these three provinces rank among the top in terms of the absolute number of higher education institutions (Jiangsu: 1/31, Anhui: 9/31, Zhejiang: 11/31). Moreover, most of these provinces show high levels of industry–education integration (Zhejiang: 1/31, Jiangsu: 2/31, Anhui: 20/31).

Configuration H2 can be termed the “Digital-Enabled Industry–Education Integration” model. A key characteristic of Configuration H2 is the balanced development of all symbiotic elements, particularly its high levels of DE and IEI. H2a and H2b exhibit extremely high similarity and are primarily represented by Tianjin, Shanghai, Fujian, and Liaoning—provinces where symbiotic elements are well-balanced and show no significant weaknesses, especially excelling in digital technology application and industry–education integration. H2c is represented by Beijing and Chongqing. Despite lagging somewhat in industrial park development, these regions have successfully established national MIECs by leveraging their strong higher education institutions and enterprises, supported by a favorable innovation environment, advanced digitalization, and industry–education integration.

Configuration H3, which we refer to as the “Assistance-Driven” model, is primarily represented by Xinjiang. Compared with other provinces, these regions exhibit relatively weak symbiotic elements, in which only industrial park is the core condition present, and higher education institutions is the edge condition present. All other symbiotic elements are core-absent, while enterprises are edge-absent. The primary reason why these provinces have succeeded in establishing national MIECs lies in the national policy on east–west collaboration and paired assistance, which affords them “special consideration” from the Ministry of Education. Concurrently, stakeholders—including local governments, industrial parks, universities, and enterprises— demonstrate strong commitment and implement targeted measures to develop high-quality MIECs.

#### 5.1.3. Mechanism analysis of non-high-level MIECs.

Failure is the mother of success. Analyzing the configurations for non-high-level MIECs helps other regions to avoid development pathways that lead to such consortia.

From the perspective of configurations, there are four configurations that give rise to non-high-level MIECs, which can be classified into three categories. The representative provinces of Configuration L1 are Tibet, Qinghai, Gansu, and Hainan. These provinces share a common characteristic: they suffer from an underdeveloped institutional environment and weak higher education systems, resulting in low levels of industry-education integration. Even though their digitalization levels may be relatively high, they are still unable to establish high-quality MIECs. Configuration L2 is represented by Inner Mongolia and Shanxi. In these regions, higher education institutions are the only core-present symbiotic element, while all other symbiotic elements are absent—particularly industrial parks and industry-education integration, both of which are core-absent. Configuration L3 is represented by Henan. In this configuration, P and IEI are core-absent, while higher education institutions and the digital environment are marginally absent. Although enterprises, the institutional environment, and the innovation environment are present, Henan has still failed to successfully establish a national MIEC.

In terms of symbiotic elements, the configurations for non-high-level MIECs share a common feature: the seven conditional variables are usually underdeveloped. Although some individual conditional variable may be developed, they are insufficient to constitute high-level MIECs. U, P, InstE, and IEI are particularly important because three or even all four of these conditional variables are absent—often as “core-absent”— in the four configurations for non-high-level MIECs.

### 5.2. Research implications

#### 5.2.1. Theoretical implications.

This paper adopts a provincial administrative region perspective to study the MIECs. It treats each PAR as a PIEIE, analyzes how PARs establish MIECs, and investigates the causal configurations associated with high-level and non-high-level MIECs. MIECs are composed of multiple symbiotic units, including industrial parks, schools, and enterprises. However, these units are different in their interests, operational approaches, and governance models, which complicates resource integration and coordinated management. As a result, it is difficult for individual symbiotic unit alone to achieve the goal of building high-level MIECs. Governments need to take the lead in formulating top-level design, coordinating the interests of all parties, and providing a sound ecosystem for the development of high-level MIECs. Many previous studies have directly focused on MIECs, examining their operational mechanisms, existing problems, and corresponding solutions, which sometimes lack operability and are difficult to implement. As MIECs are policy-driven initiatives, studying them from the perspective of provincial administrative regions yields findings with greater practical applicability.

The fuzzy-set Qualitative Comparative Analysis (fsQCA) method is adopted in this study for three main reasons. First, fsQCA is well-suited for studies with small samples, making it appropriate for this study’s analysis of 31 cases. Second, prior studies have predominantly relied on qualitative approaches to identify the problems associated with MIECs and propose corresponding solutions. In contrast, fsQCA combines the qualitative focus on in-depth case analysis while incorporating the systematic rigor and reliability of quantitative analysis. Third, by adopting a configurational perspective at the provincial level to investigate the pathways to high-level MIECs, this study addresses the limitations of conventional statistical methods, which typically examine symbiotic elements in isolation rather than as interconnected configurations.

Based on symbiosis theory, this study analyzes the configurational paths through which the symbiotic elements of the PIEIE contribute to the establishment of high-level MIECs. It is found that the establishment of high-level MIECs is not determined by a single factor, but by the synergistic effect of multiple factors. There are 6 configurational paths leading to the establishment of high-level MIECs, which can be categorized into three types: the “Economy-Driven” model, the “Digital-Enabled Industry–Education Integration” model, and the “Assistance-Driven” model.

#### 5.2.2. Managerial implications.

MIECs are policy-driven entities, significantly influenced by local industrial and educational development as well as government policies. Studying the construction of high-level MIECs within a province from the perspective of the PIEIE can provide policymakers with theoretical guidance and practical evidence.

This study not only compares the number of MIECs across the 31 PARs, but also evaluates the development of high-level MIECs adjusted for regional scale, thereby offering a more comprehensive assessment of regional progress.

Drawing on the distinctive features of the PIEIEs, the regions should seek context-specific pathways to develop high-level MIECs.

Configuration H1, named the “Economy-Driven” model, which leads to the formation of high-level MIECs, requires high levels of innovative environment, institutional environment, and industrial parks, supported by strong performance in enterprise engagement and industry-education integration—even when higher education development is relatively low. Configuration H1 provides valuable experience for other major economic provinces, such as Henan. Its rankings on key symbiotic elements are as follows: U(27/31), E(15/31), P(24/31), IE(9/31), InstE (15/31), DE(31/31), IEI(29/31). Overall, Henan lags behind in higher education, industrial parks, digital infrastructure, and industry-education integration. Compared with the requirements of Configuration H1—the “Economy-Driven” model—Henan’s performance in industrial park development and industry-education integration falls notably short. Provinces with similar profiles should strengthen industrial parks and industry-education integration to align more closely with Configuration H1.

Configuration H2 is characterized by high performance across almost all symbiotic elements, particularly digital environment and industry-education integration, which act as indispensable core components. Configuration H2 offers valuable insights for provinces such as Hainan and Gansu. Taking Hainan as an example, its rankings on key symbiotic elements are as follows: U (16/31), E (20/31), P (23/31), IE (5/31), InstE (15/31), DE (5/31), and IEI (17/31). Overall, Hainan performs relatively well in innovative environment and digital environment, while the other symbiotic elements rank at a moderate level. Compared with the requirements of Configuration H2, Hainan’s level of industry-education integration remains insufficient. Provinces with similar profiles should holistically enhance their symbiotic elements, with particular emphasis on strengthening industry-education integration.

Configuration H3 places strong emphasis on industrial parks and higher education, while other symbiotic elements are not consistently present at high levels. Configuration H3 is unique and lacks broad generalizability; however, autonomous regions and provinces receiving targeted assistance—such as Tibet, Inner Mongolia, and Ningxia—may still draw valuable lessons from it. Given their limited resources, these provinces should prioritize the development of high-level MIECs through national paired-assistance mechanisms, with local governments taking the lead in identifying key MIECs and strategically allocating scarce resources.

The PARs should strengthen the foundations of their industry-education integration ecosystems to avoid pathways that lead to non-high-level MIECs. Non-high-level MIECs are not caused by any single symbiotic element in isolation, but rather emerge from the combined underperformance of multiple elements. The PARs should therefore avoid the four low-level configurations identified in this study. Particular attention should be paid to strengthening the four symbiotic elements—IEI, U, P, and InstE—as these collectively represent the core deficient elements across the four pathways that lead to non-high-level MIECs outcomes.

## 6. Conclusions, limitations and future research

### 6.1. Conclusions

MIECs are a key initiative in China’s vocational education reform, playing a vital role in talent development, innovation and entrepreneurship, and the high-quality growth of regional industries. As a policy-driven construct, MIECs are deeply shaped by their environment. At the provincial level, each province constitutes a symbiotic system of industry-education integration, within which MIECs emerge and evolve. To examine how the symbiotic elements of the PIEIEs influence the formation of high-level MIECs, this paper draws on symbiosis theory to develop a conceptual model linking provincial symbiotic systems to high-level MIECs outcomes. It employs fsQCA to identify the configurational pathways that lead to high-level MIECs.

This study examines the development of high-level MIECs across China’s 31 provinces, autonomous regions, and municipalities—excluding Hong Kong, Macao, and Taiwan—and finds that the emergence of high-level MIECs is not driven by any single symbiotic element in isolation, but rather results from the synergistic interaction of multiple elements. There are six configurational pathways leading to high-level MIECs, grouped into three distinct types, and four pathways associated with low-level MIECs, classified into three types. These findings provide both theoretical grounding and practical guidance for the PARs to design context-sensitive industry-education integration ecosystems tailored to their unique conditions in order to foster high-level MIECs.

Overall, this paper provides a new perspective for the research on MIECs and enriches the relevant research methodologies. The findings of this paper offer theoretical basis and practical guidance for the PARs to create an industry-education integration ecosystem conducive to fostering high-level MIECs.

### 6.2. Limitations and future research

First, this study focuses exclusively on MIECs in China. While similar consortia of cross-sectoral collaboration between education and industry exist in other countries, their institutional, economic, and policy environments differ significantly. Future research could extend comparative analyzes to both developed and developing countries, investigating how MIECs—or their functional equivalents—are shaped under diverse configurations of policy frameworks, economic conditions, industrial structures, educational systems, and levels of industry-education integration.

Second, the analytical perspective adopted in this paper is static, relying on cross-sectional data. However, the development of MIECs inherently involves temporal dynamics and lag effects—for instance, investments in institutional capacity or ecosystem building may only yield outcomes after a certain time lag. To address this limitation, future studies could employ panel data and incorporate time-lagged variables to better capture the dynamic influence of symbiotic ecosystem elements on the formation and evolution of MIECs within regional contexts.

## Supporting information

S1 File Original case data. (XLSX)

## Abbreviations

PAR: provincial-level administrative region
MIEC: municipal industry-education consortium
fsQCA: fuzzy set qualitative comparative analysis
RIEIE: regional industry-education integration ecosystem
PIEIE: provincial-level industry-education integration ecosystem
U: University
E: Enterprise
P: Industrial park
IE: Innovation environment
InstE: Institutional environment
DE: Digital environment
IEI: Industry-education integration
